# Multiple disc herniation in spondyloepiphyseal dysplasia tarda: A rare case report and review of the literature

**DOI:** 10.1186/s12891-022-06064-4

**Published:** 2022-12-13

**Authors:** Zan Chen, Zheyi Zhang, Fei Ye, Fei Lei, Daxiong Feng

**Affiliations:** 1 Department of spine surgery, The Affiliated Hospital of Southwest Medical University, No 25 TaiPing St, Jiangyang District, Luzhou, Sichuan 646000 People’s Republic of China; 2 Department of finance, The Affiliated Hospital of Southwest Medical University, Luzhou, Sichuan People’s Republic of China

**Keywords:** Spondyloepiphyseal dysplasia tarda, Spondyloepiphyseal dysplasia tarda with progressive arthropathy, Progressive pseudorheumatoid dysplasia, Spinal disorder, Surgical intervention

## Abstract

**Background:**

Spondyloepiphyseal dysplasia tarda (SEDT) is a rare, hereditary, X-linked skeletal disorder. To our knowledge, there are few reports about orthopedic surgery in these patients. This is the first report on patients with SEDT undergoing spinal and fracture reduction surgery.

**Case presentation:**

A 31-year-old male patient who had been misdiagnosed with juvenile idiopathic arthritis (JIA) for 20 years and who had been treated with femoral shaft internal fixation for lower extremity fracture caused by minor trauma presented at hospital with stiffness and weakness in the lower extremities for the past two years. Radiographs showed bony dysplastic features of flattened vertebral bodies, Scheuermann-like changes in the spine, and osteoarthritis-like changes in the joints. Laboratory examination, including routine blood tests and rheumatism-related indicators showed negative results. Considering the history, radiology, and genetic findings, a diagnosis of spondyloepiphyseal dysplasia tarda with progressive arthropathy (SEDT-PA) was considered. Further neurological examination indicated that severe spinal cord compression was an important reason for the patient’s inability to walk. Laminectomy, spinal canal decompression, internal fixation and fusion were performed. Clinical outcome was satisfactory at one-year follow-up. The lower-limb fatigue was relieved, the patient could walk independently, and his examination showed osseous fusion. The English database was searched and the literature was reviewed for the relevant keywords of “SEDT-PA”.

**Conclusions:**

Progress has been made in genetic research on SEDT; early diagnosis is particularly important, but the clinical diagnosis and treatment plans are still evaluated on a case-by-case basis. The best treatment for SEDT is to identify patients with progressive neurological and joint-mobility impairments and perform appropriate surgical intervention. Surgical intervention can improve neurological function and quality of life. However, surgery, as palliative care, does not alter the progression of the disease.

## Background

Spondyloepiphyseal dysplasia tarda (SEDT) is a rare autosomal recessive osteochondroplasia [1, 2]. Growth is normal at birth, however joint pain and swelling may begin to occur around 3–6 years of age [2]. The disease primarily affects the articular cartilage and is characterized by progressive joint stiffness and enlargement. This causes disorders of articular cartilage bone formation, premature osteoarthritis, and cartilage degeneration, and the attacks are symmetrical [3]. However, the disease was misdiagnosed as juvenile idiopathic arthritis (JIA) in adolescence, and early patients were prescribed unnecessary anti-inflammatory and antirheumatic drugs [4]. We report the first case of SEDT patients undergoing spinal decompression fusion and fracture internal fixation surgery.

### Case presentation

**Fig. 1:**
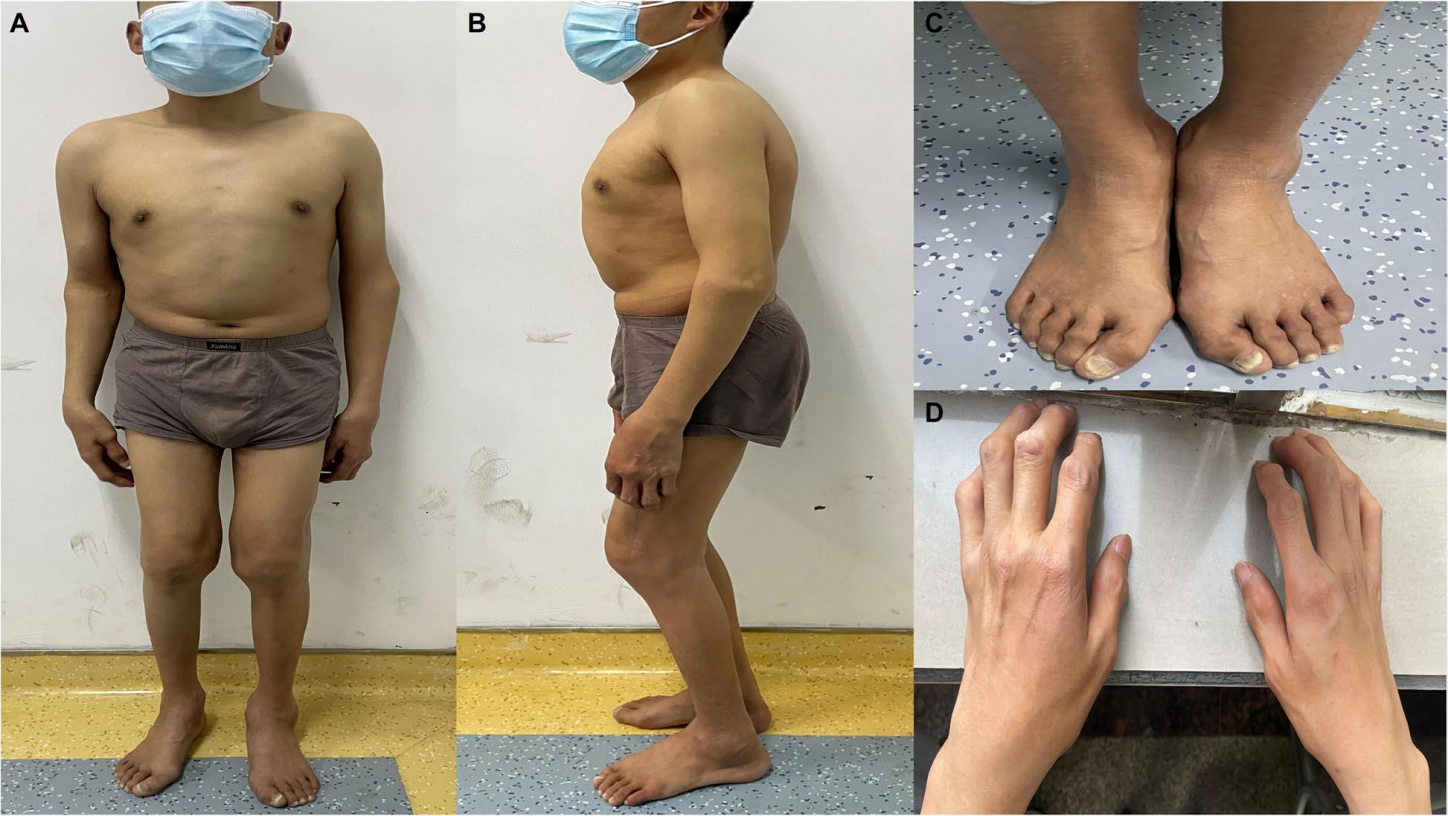
In terms of overall appearence (**a, b**): short stature, barrel chest deformity, anterior flexion of the spine, humpback deformity, and multiple muscle atrophy. Increased swelling of the interphalangeal joints of the hands and feet (**c, d**)

A 31-year-old male proband presented with back pain accompanied by weakness and numbness in both lower limbs. The proband’s parents were not consanguineous, and the mother was not exposed to radiation or toxic chemicals during pregnancy. There were no similar symptoms or signs in the proband’s family. The patient had been delivered at term and was within the normal ranges for height and weight at birth. Signs of his illness first appeared at age 5 years, with obviously enlarged finger and toe joints. At the age of 10, he had bilateral knee pain and was unable to walk normally. At that time, he was shorter than his peers, and he received no more schooling. At the age of 12, he was admitted to a local hospital. The diagnosis of JIA was considered, and he received anti-rheumatic drug treatment for a long period, developing limited mobility of the limbs. Five years prior to the current visit, after a minor trauma, the patient’s femoral shaft was fractured, and after 2 years of internal fixation of the lower extremity, he was able to walk without assistance. Two years ago, he developed numbness and weakness in both lower limbs, and over the past 6 months. He experienced progressive aggravation of muscle rigidity, necessitating the use of crutches when walking. Physical examination indicated that he was 132 cm tall, weighed 46 kg, had an arm span of 130 cm, and was 66 cm tall in the seated position. In appearance, he had a short stature, a barrel chest deformity, forward flexion of the spine and a posterior hump, and atrophy of several important muscle groups (Fig. 1a, b). His facial features and intellectual development were normal. The patient’s shoulder, wrist, knee, and ankle joints were symmetrically limited, and significant swelling in the interphalangeal joints of his hands and feet was observed (Fig. 1c, d). He exhibited thoracic scoliosis and limited mobility of the cervical and lumbar spine. Neurological examination revealed spastic paralysis, hypoesthesia below the T10 plane, decreased hip flexion and knee extensor strength, hyperactive bilateral knee reflexes, ankle contractures, undetectable Achilles tendon reflexes, and a positive Babinski sign. The erythrocyte sedimentation rate (12 mm/h) and C-reactive protein (2 mg/h) were within the normal range, and rheumatoid factor was negative. Dual-energy x-ray examination showed a minimum Z-score of − 3.8 for bone mineral density. The visual analog scale (VAS) score was 8, and the Oswestry disability index (ODI) score was 66.

**Fig. 2:**
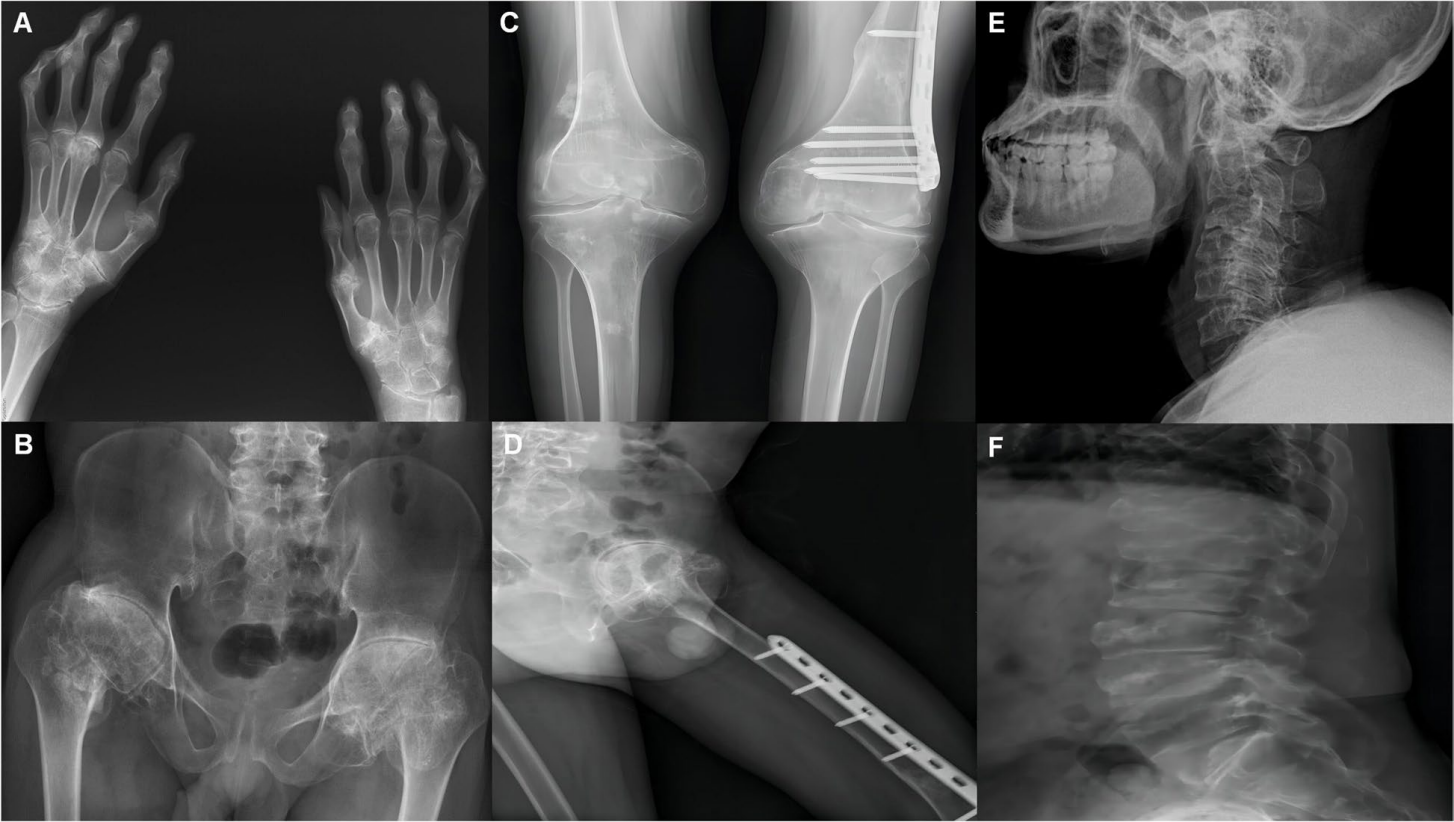
Hand and pelvis x-rays **(a, b)** showed increased light transmittance, narrowing of the joint space, hyperostosis and sclerosis of the subarticular surface, and bony enlargement. Bilateral femoral neck shortening, discontinuous calve line. The left femur **(c, d)** was fixed in place, the bone structure of the lower femur was disordered, and callus formed. Cervical and lumbar **(e, f)** decreased bone mineral density, vertebral bodies were compressed and flattened to varying degrees, marginal osteophytes were formed, and endplates were irregular

**Fig. 3:**
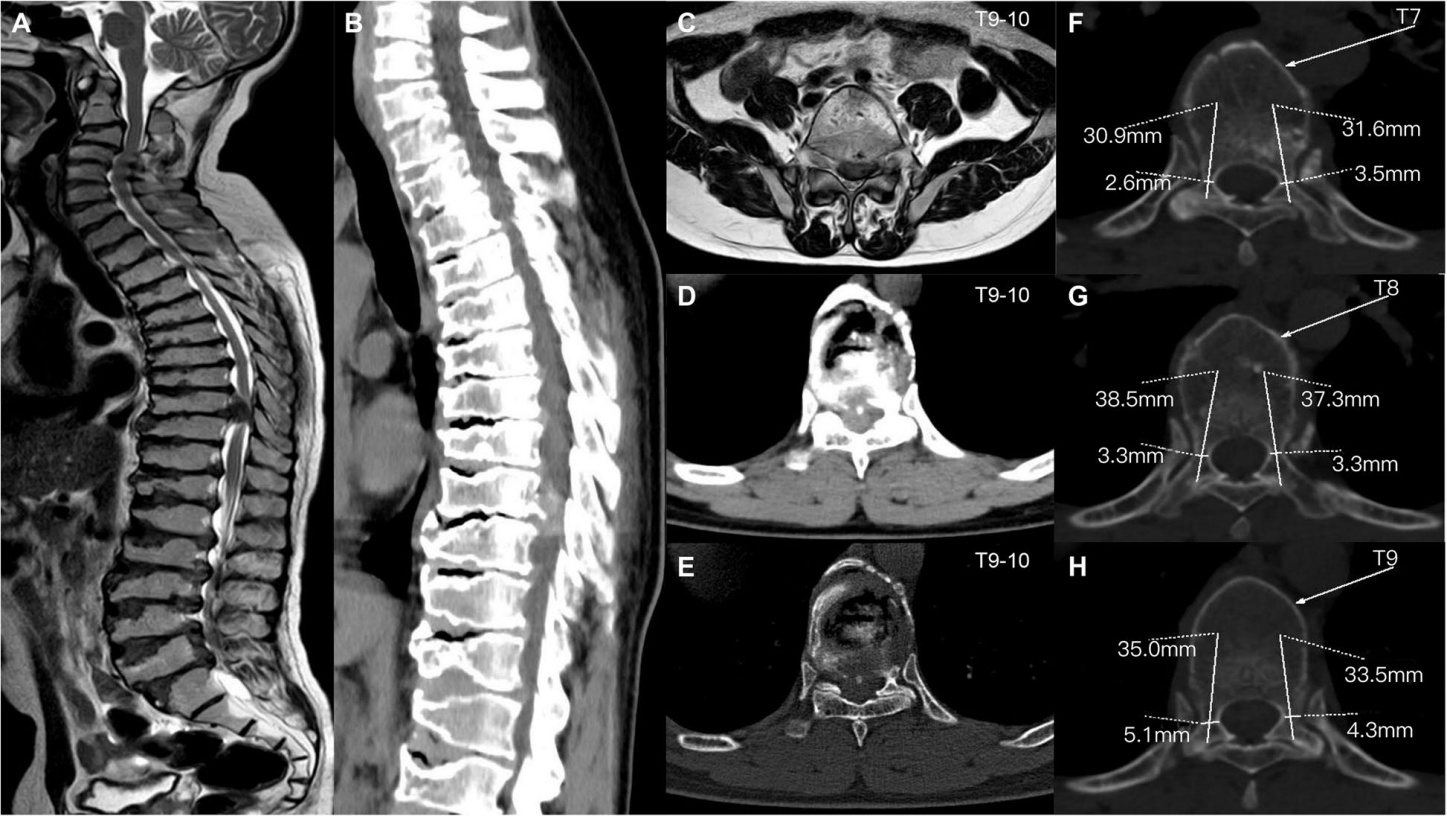
Preoperative T2-weighted magnetic resonance imaging (MRI) and computed tomography (CT) sagittal scan **(a, b)** showed multiple discs with reduced signal (intervertebral disc vacuum phenomenon) and spinal stenosis. Transverse view **(c, d, e)** of the intervertebral disc at T9–10 suggested posterior inferior disc prolapse with spinal cord compression. Measurements on a CT transverse view **(f, g, h)** of the pedicle suggested a small pedicle

**Fig. 4:**
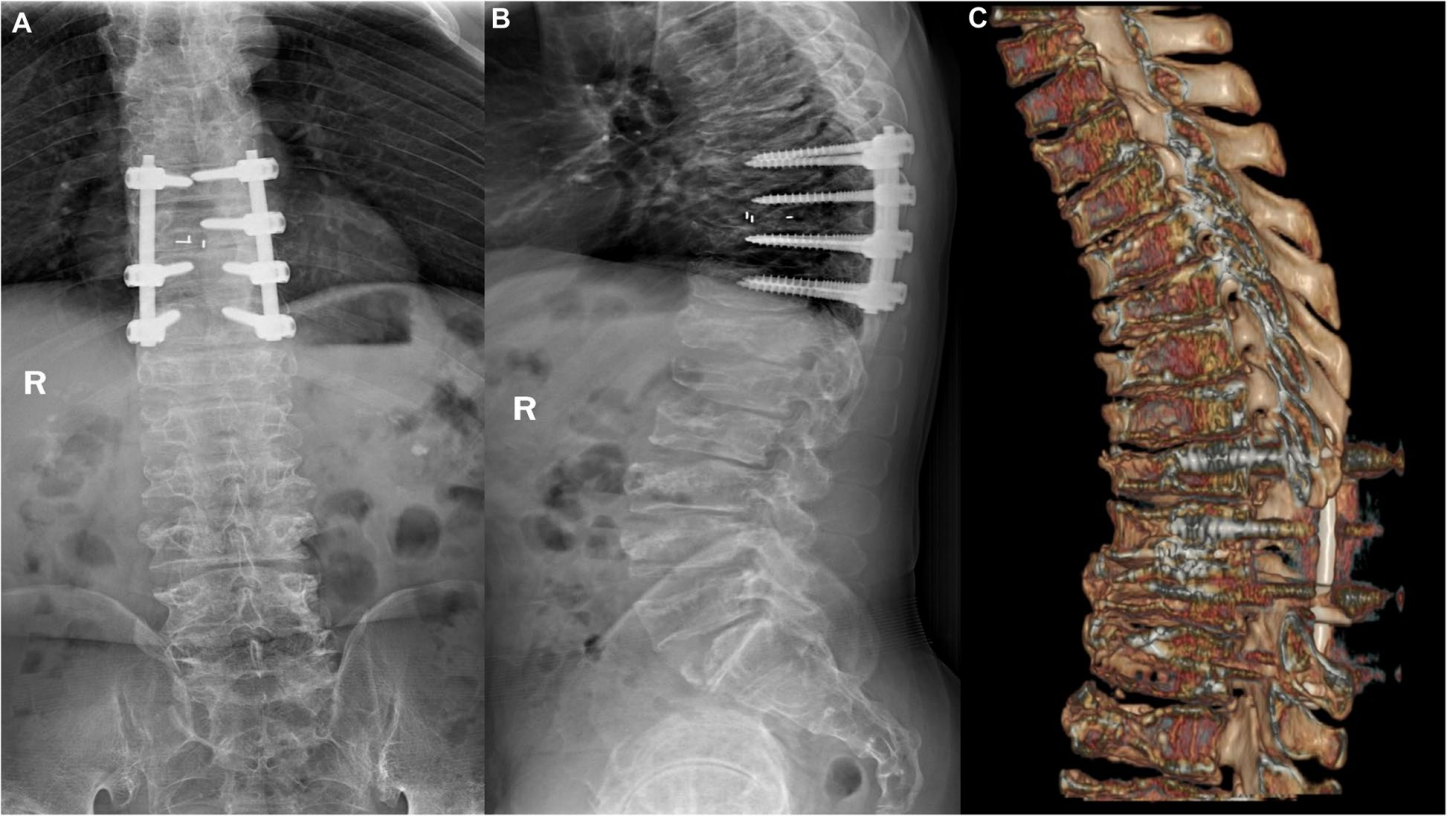
The one-year follow-up X-ray and three-dimensional CT **(a, b, c)** showed that T8–11 internal fixation was in place, T9–10 intervertebral high density shadow

Radiographic examination of the patient’s hands revealed an enlarged and widened epiphysis and narrowing of the joint space (Fig. 2a). Pelvic radiographs showed an enlarged proximal femoral epiphysis on both sides, an irregular femoral head contour, and diffuse osteoporosis (Fig. 2b). Plain radiographs of the lower extremities showed that the femur was fixed in place and the bone had healed well (Fig. 2c, d). Spinal radiographs showed flat vertebral bodies, prominent dorsal humps, irregular endplates, and short pedicles (Fig. 2e, f). Three-dimensional CT and MRI of the spine showed that T9–10 giant hernia accounted for 70% of the medullary canal area (Fig. 3c, d, e), different degrees of herniation in L1-S1, and multi-segment “vacuum phenomenon” manifestations (Fig. 3a, b). The patient did not have lower-extremity radiating pain or intermittent claudication. Excluding Intracranial and cervical spine diseases, the symptoms of lower extremity spastic paralysis were considered to be mainly caused by thoracic spinal cord compression. T9–10 total laminectomy through the posterolateral approach was used to enter the intervertebral space, and bilateral dural hernias were isolated under the direct microscope. After the anterior compression of the thoracic cord was relieved, the fluctuation of dural congestion was satisfactory. Due to the destruction of the posterior bone structure and the removal of the intervertebral disc tissue, internal fixation with T8–11 screws and fusion with a T9–10 cage (Kangsheng, DFII, Chinese Inc.) were performed. Intraoperative evidence of premature degenerative changes in spinal attachment and intervertebral discs, while the development of small pedicles, severe degeneration led to aggravation of the adhesion of the anterior side of the dural sheath to the hernia. Postoperative lower limb sensation and muscle stiffness were significantly improved compared with preoperative values, but there was no significant improvement in muscle strength. Before discharge, we recommend long-term anti-osteoporosis therapy with bisphosphonates due to poor bone quality. The patient’s muscle strength was recovered 6 months after the operation, and walking assistance was not needed . At the one-year follow-up after the operation, three-dimensional CT scan showed satisfactory spinal canal decompression and osseous fusion (Fig. 4a, b, c). The one-year follow-up VAS score was 3 points, and the ODI score was 42 points.

## Discussion and conclusion

SEDT was first reported by Wynne-Davies in 1982 [5]. The exact incidence of SEDT in the population is unknown, and most cases remain undiagnosed, with an estimated incidence of only 1 in 1 million in the UK [5]. Males are predominately affected. Such patients are born with normal growth, subsequently (at age 3–6 years) developing joint pain and joint swelling, along with nonsynovial swelling and bilateral joint involvement [2]. SEDT begins in the interphalangeal joints with progressive development in large joints along with spinal cord involvement. This causes serious consequences such as movement disorders, joint contractures, scoliosis, and nerve compression. In the present case, the first symptoms appeared in early childhood, and the patient was referred to the rheumatology department for investigation [6]. Negative inflammatory markers were misdiagnosed as JIA [7]. A diagnosis of SEDT is usually made only in the second decade [2], often by orthopedic physicians due to the painful symptoms, and neurological symptoms are thought to appear after the third decade [3]. Raising awareness of SEDT appears to be critical for timely diagnosis.

There are two forms of SED in patients. One is congenital SED, which occurs at birth and is caused by genetic mutations in COL2A1 [8]. The other is SED tarda, which appears in childhood and is divided into X-linked and autosomal recessive depending on the genotype [6, 9, 10]. The cause of X-linked SEDT is the TRAPPC2 gene mutation. The interaction of TRAPPC2, which localizes to xp22, and multiple transcription factors might regulate the expression of genes involved in skeletal development [10]. Autosomal recessive inheritance, also known as spondyloepiphyseal dysplasia tarda with progressive arthropathy (SEDT-PA), results from mutations in the WISP3 gene. The gene is located on chromosome 6q22. WISP3 belongs to the CCN family of stromal cell proteins that control cell differentiation and proliferation, as well as angiogenesis, chondrogenesis and osteogenesis [6, 9]. In addition to the difference in genotype, whether or not osteoarthritis involving the peripheral joints is also one of the identification methods [11].

In the case reported here, the patient’s family tree extending for nearly three generations did not show any signs of SEDT. We suspect that there may be two genetic types. The patients and their families signed informed consent, and the WISP3 gene detection further confirmed the diagnosis of SEDT-PA. Gene sequencing revealed compound heterozygous mutation: NM_198239.1 (WISP3): c.1064_1065dupGT and c.643 + 2 T > C. Mutation types were mentioned in previous reports [12]. Patients with SEDT-PA have similar cartilage-damage characteristics across genotypes, but there is no precise relationship between mutation type and phenotypic severity. Use descriptors (progressive pseudorheumatoid dysplasia OR progressive pseudorheumatoid arthropathy of childhood OR spondyloepiphyseal dysplasia tarda with progressive arthropathy OR SEDT-PA OR spondyloepiphyseal dysplasia tarda [Title/Abstract]) AND (surgery or replacement or arthroplasty [text word]) reviewed the Pubmed database and searched for relevant articles from 1990 to the present. For included cohorts with orthopedic surgery interventions in the test, only patients with SEDT-PA were included. At the same time, relatively complete clinical, genetic and prognostic data were included in the review. Of the 34 articles retrieved, 10 articles were finally included (Table 1). Fifteen of 15 patients underwent orthopedic surgery, including 9 lower extremity surgery, 4 spine surgery, and 2 combined surgery, of which 10 had evidence of mutation. There are currently no reports on specific mutational subtypes in this group of patients, and each patient received individualized treatment (Table 1).

**Table 1:** Characteristics of included studies

Author,Year	Sex	Age	Symptoms	Bone problems	Gene mutation/ Nucleotide Change	Surgery/complication	final outcome
Feng et al. [13] 2021	Male	23	Hip pain and stiffness	EFH, NNJ	WISP3/c.1064G > A	THA	Pain relief,restore joint function
	Female	17	Hip pain and stiffness	EFH, NNJ	WISP3/c.670dupA	THA	Pain relief,restore joint function
	Female	29	Hip pain and stiffness,mild flexion deformity of knees	EFH, NNJ, EFC	WISP3/c.670dupA	THA/screw impinging onto the sciatic nerve,remission after revision surgery	Pain relief,restore joint function
	Male	26	Hip pain and stiffness,mild valgus knee deformity	EFH, NNJ, EFC	WISP3/c.670dupA	THA	Pain relief,restore joint function
	Male	26	Lower limb incomplete paraplegia	PY, KY	**–**	SD,PSF	Neurologically improved
	Female	13	Hip and knee flexion contracture	EFH, NHJ, EFC, NKJ	**–**	Ilizarov technique	Restore joint function
Li et al. [14] 2019	Male	35	Lower limb incomplete paraplegia associate with radiculopathy	PY, KY	WISP3/c.395G > A/p.C132Y	SD,PSF	Neurologically improved but muscle weakness did not improve
Yang et al. [3] 2013	Male	44	Pain in the left hip;lower limbs sciatica,hypoesthesia and hypermyotonia	EFH, NHJ, PY, KY	WISP3/c.840delT	THA,SD,PSF /Cerebrospinal fluid leakage	Neurologically improved，osseous fusion
Ikegawa et al. [15] 1993	Female	32	Bilateral coxalgia and waddling gait	EFH, NHJ	–	THA	Restore joint function
Nakamura et al. [16] 1993	Male	19	Lower limb incomplete paraplegia associate with radiculopathy	PY, KY,NHJ	–	SD	Neurologically improved
Gao et al. [17] 2013	Female	17	Hip pain and stiffness	EFH, NHJ	–	THA	Pain relief, restore joint function
Xiao et al. [12] 2018	Male	17	hip and knee pain and stiffness	EFH, NHJ, EFC, NKJ	WISP3/c.1064_1065dupGT and c.643 + 2 T > C	Ilizarov technique	Restore joint function
Zhou et al. [18] 2007	Female	19	Back pian,hip pain and stiffness	EFH, NHJ,PY	WISP3/c.840delT and 1000 T > C	THA	Restore joint function
Cassa et al. [19] 2016	Male	34	Hip and knee flexion contracture,back pain	EFH, NHJ, EFC, NKJ, PY	WISP3/p.Cys265LeufsX31	THA,TKA,SD	Restore joint function
current study 2022	Male	31	Back pain,weakness and numbness in lower limbs	PY, KY,EFH, NHJ	WISP3/c.1064_1065dupGT and c.643 + 2 T > C	SD,PSF	Pain relief,neurologically improved，osseous fusion

*EFH* enlargement of femoral heads, *NHJ* narrowing hip joint space, *EFC* enlargement of femoral condylar, *NKJ* narrowing knee joint space, *PY* platyspondyly, *KY* kyphosis, *THA* total hip arthroplasty, *TKA* total knee arthroplasty; *SD* spinal decompression, *PSF* posterior spinal fusion

We found that these patients had different genotypes and phenotypes, different clinical phenotypes, different degrees of disease progression and different treatment prognoses [12, 13]. Due to the premature appearance of nerve damage and joint mobility impairment, orthopedic surgery is required in adolescence.

There are more patients with SEDT-PA undergoing lower extremity surgery [3, 12, 13, 17–19]. Because dysplasia affects bone growth and cartilage homeostasis, joint dysfunction in weight-bearing joints occurs in adolescence [13, 20]. Joint replacement surgery is an inevitable final option [2, 3, 17]. Gao et al. [17] believe that it is necessary to delay the timing of joint replacement surgery as much as possible after closing the lower extremity epiphysis. For patients with joint contractures, Ilizarov external fixation apparatus allows passive movement of major joints to release soft tissue [12]. As a way to delay artificial replacement surgery. Feng et al. [13] conducted a mid-term follow-up of 8 confirmed patients who received the artificial joint replacement, with good improvement in pain and functional activity. Hip dysplasia combined with osteoporosis increases the difficulty of surgery. The treatment of deformities and contractures of the elbow and interphalangeal joints in the upper extremities is still unknown.

For the need for spinal surgery, neurological deficits are the primary consideration, especially for those with neurological deterioration [21]. As early as 1998, Nakamura et al. [16] reported that a man engaged in heavy labor underwent two-time laminectomy decompression surgery due to multiple herniated discs, and his preoperative symptoms were significantly improved. For the simultaneous presence of osteoporosis and kyphosis, simple spinal decompression may not achieve good spinal stability, and internal fixation and fusion surgery are considered safe and effective [14]. At the same time, prolonging the internal fixation segment can yield good stability. Satisfactory recovery was achieved in two other reports of spinal fusion for pseudorheumatoid dysplasia [3, 14]. Preoperative screw planning is necessary for pedicle dysplasia which is also safe and effective for interbody fusion (Fig. 3f, g, h). During follow-up, bone healing in this and another article was followed at one year, and no loosening or broken screw occurred, but long-term follow-up was lacking [3]. Achondroplasia and osteoporosis are considered risk factors that may affect bone healing [2, 22]. Due to delayed healing of the femoral shaft fracture due to low-energy trauma, the internal fixation has not been removed to date. This is the first reported case of patients with SEDT with severe spinal degeneration and poor bone quality undergoing lower extremity and spine surgery. Fragility fractures have led us to realize the early development of osteoporosis in these patients. The mechanism of osteoporosis is still unclear, and it may be related to defective bone mineralization, and disuse osteoporosis related to joint disorders cannot be ignored [17, 23]. The role of anti-osteoporotic and nonsteroidal drugs in these patients is unclear. Based on a prior low-energy fracture, the patient was diagnosed with osteoporosis, and we still recommend bisphosphonates based on general consensus [24]. WISP3 regulates the expression of extracellular matrix proteins in chondrocytes, therefore mutations of WISP3 affect the spinal cartilage endplates and annulus fibrosus. Early appearance of endplate collapse, annulus fibrosus rupture, axial pain caused by short spine, imbalance, and nerve damage need to be paid more attention to. In this article, imaging suggests multiple lumbar disc herniations without neurogenic symptoms, which is necessary for this patient’s follow-up whether underwent surgical intervention is required.

In conclusion, the diagnosis of SEDT is based on the clinical phenotype, imaging and genetic diagnosis. Although SEDT does not affect a patient’s life expectancy, the neurologic deficits and impairment of joint mobility significantly affect quality of life, and appropriate surgical intervention is effective. There is still a lack of long-term follow-up observations for orthopedic surgery, and therapeutic interventions for the different joint deformities remain to be discovered.

## Data Availability

All relevant data was presented within the manuscript and the datasets used and/or analyzed during the current study are available from the corresponding author on reasonable request.

## Abbreviations

SEDT: Spondyloepiphyseal dysplasia tarda
SEDT-PA: Spondyloepiphyseal dysplasia tarda with progressive arthropathy
JIA: Juvenile idiopathic arthritis
VAS: Visual analog scale
ODI: Oswestry Disability Index
CT: Computed tomography
MRI: Magnetic resonance imaging
EFH: Enlargement of femoral heads
NHJ: Narrowing hip joint space
EFC: Enlargement of femoral condylar
NKJ: Narrowing knee joint space
PY: Platyspondyly
KY: Kyphosis
THA: Hip arthroplasty
SD: Spinal decompression
PSF: Posterior spinal fusion.
